# Novel Targets and Small Molecular Interventions for Liver Cancer

**DOI:** 10.1155/2014/148783

**Published:** 2014-08-04

**Authors:** Chunping Jiang, Youmin Wu, Jian Zhou, Jingmin Zhao

**Affiliations:** ^1^Department and Institute of Hepatobiliary Surgery, The Affiliated Drum Tower Hospital, Medical School, Nanjing University, Nanjing, Jiangsu 210008, China; ^2^Intra-Abdominal Transplant and Hepatobiliary Surgery, Westchester Medical Center of New York Medical College, Valhalla, NY 10595, USA; ^3^Department of Liver Surgery and Liver Cancer Institute, Zhongshan Hospital, Fudan University, Shanghai 200032, China; ^4^Department of Pathology and Hepatology, Beijing Institute of Infectious Diseases, Beijing 302 Hospital, Beijing 100039, China

Liver cancer is currently the fifth most common solid tumor worldwide, causing almost 7000 deaths every year. Among primary liver cancers, hepatocellular carcinoma (HCC) represents the major histological subtype, accounting for 70% to 85% of the total liver cancer burden. To date, the major etiologies and risk factors for liver cancer development are well-defined. Due to the progress in clinical and biological fields, the primary carcinogenetic steps and molecular mechanisms have been elucidated in recent decades. The interventions for liver cancer patients vary depending on the stages. Surgical resection remains the most effective method for liver cancer patients of early stage. For patients of advanced stages, palliative therapies such as percutaneous ethanol injection, radiofrequency ablation, microwave ablation, transarterial chemoembolization (TACE), selective internal radiation therapy (SIRT), and sorafenib are recommended treatment choices for unresectable liver cancers. Despite the advances in diagnostic and therapeutic measures, the prognosis of liver cancer is not satisfying, with the 5-year survival rate being less than 12%. So the discovery of new interventions is still in demand. Recently, much attention has been paid to exploring new molecular mechanisms that might be involved in liver cancer biological behaviors. We believe further study of novel targets and small molecular interventions would be helpful to improve the prognosis of liver cancer.

In this current issue, we focus on recent advances in the field of novel targets and small molecular interventions for liver cancer which might help reveal the possible mechanism of tumorigenesis, progression, metastasis, and recurrence of liver cancer and contribute to emerging therapeutics for liver cancer. We present nine articles on novel targets and small molecular interventions for liver cancer of which six investigate the targets and mechanisms for hepatocarcinogenesis, progression, metastasis, recurrence, and HCC drug resistance, two introduce novel agents for HCC treatments, and one makes a comprehensive review on the novel molecular targets for future therapies of HCC.

The paper titled “*Reexpression of let-7g microRNA inhibits the proliferation and migration via K-Ras/HMGA2/snail axis in hepatocellular carcinoma*” by K. Chen et al. found that reexpression of let-7g inhibited the proliferation, migration, and invasion of HCC, and low expression of let-7g was significantly associated with poorer overall survival.

The work by H. Xiao et al. investigated BAG3 and HIF-1*α* expression in HCC tissues and analyzed the association between BAG3 and HIF-1*α* coexpression and prognosis following liver transplantation. They found that expression level of BAG3 and HIF-1*α* was efficient prognostic parameters in patients with HCC after liver transplantation.

The work by Z. Wang et al. analyzed the association between two common polymorphisms (miR-146a G>C and miR-196a2 C>T) and risk of HCC by meta-analysis. MiR-146a G>C and miR-196a2 C>T were associated with decreased HCC susceptibility, especially in Asian population.

The work by Y.-X. Liu et al. analyzed the role of microRNA-24 in HCC related to aflatoxin B1 and revealed miR-24 was upregulated in HCC tumor tissues. MicroRNA-24 overexpression modified the recurrence-free survival and overall survival of HCC patients. The joint effects between miR-24 and AFB1 exposure on HCC prognosis were also observed.

The work by W. Chen et al. investigated tumor microenvironment on HCC cell's reaction to sorafenib. The oral multi-tyrosine kinase inhibitor sorafenib is the only approved systemic therapy for HCC patients in BCLC stage C with significant survival benefit. In this paper, Chen revealed that hepatic stellate cell- (HSC-) LX2 coculture induced sorafenib resistance in Huh7 through HGF/c-Met/Akt pathway and Jak2/Stat3 pathway which gave support to the theory that tumor microenvironment confers drug resistance to kinase inhibitors.

C. Peng et al. studied the function of tumor suppressor ZDHHC2 in HCC. Loss of heterozygosity on ZDHHC2 was associated with early metastatic recurrence following liver transplantation. Restoration of ZDHHC2 inhibited HCC proliferation, migration, and invasion.

In the work by Z. Wang et al., baicalein was found to exhibit prominent anti-HCC activity. This flavonoid induces apoptosis and protective autophagy via ER stress. Combination of baicalein and autophagy inhibitors may represent a promising therapy against HCC.

The work titled “*Dehydroabietic acid derivative QC2 induces oncosis in hepatocellular carcinoma cells*” by G. Zhang et al. investigated the inhibitory effect of a new dehydroabietic acid derivative QC2 on HCC. They found that QC2 induced HCC cell death by oncosis through activating oncosis related protein calpain.

The review article “*Hepatocellular carcinoma: novel molecular targets in carcinogenesis for future therapies*” by G. Bertino et al. made a general review on novel molecular targets in carcinogenesis for HCC.

In summary, development of novel systemic therapies for advanced liver cancer, including drugs, small molecular agents, and gene therapies, is of paramount importance. This special issue presents several intriguing achievements in the area of novel targets and small molecular interventions which we believe could be utilized in liver cancer therapy in the future.

